# Supramolecular Zwitterionic Polymers: Dynamic Traits Imparted by Ionic Interactions

**DOI:** 10.1021/jacsau.5c01400

**Published:** 2025-12-11

**Authors:** Jin Wang, Xuedong Xiao, Xinghuo Xiao, Jianwei Sun, Ryan T. K. Kwok, Jacky W. Y. Lam, Ben Zhong Tang

**Affiliations:** † Department of Chemistry, Hong Kong Branch of Chinese National Engineering Research Center for Tissue Restoration and Reconstruction, Department of Chemical and Biological Engineering, and State Key Laboratory of Nervous System Disorders, 58207 The Hong Kong University of Science and Technology, Clear Water Bay, Kowloon, Hong Kong 999077, China; ‡ Guangdong Basic Research Center of Excellence for Aggregate Science, School of Science and Engineering, The Chinese University of Hong Kong, Shenzhen, Guangdong 518172, China

**Keywords:** ionic interactions, dynamic transition, ionic aggregates, supramolecular zwitterionic polymers, stimuli-responsive

## Abstract

Supramolecular ionic polymers (SIPs) exhibit distinctive dynamic properties, which arise from the synergistic combination of substantial strength and pronounced reversibility of ionic interactions. However, conventional SIPs formed by coassembly using anionic and cationic separated double monomers suffer from complicated synthesis and heterogeneous structures, which obscure in-depth investigation of their dynamic behaviors. In response, we developed a zwitterionic monomer, namely TPE-2N2S, that incorporates both charged motifs into one fluorescent tetraphenylethylene (TPE) skeleton. This zwitterionic and single-component monomer design strategy enables the efficient formation of supramolecular zwitterionic polymers (SZIPs) with a uniform and orderly structure, and it can further enhance the understanding of structure–property relationships. In this perspective, we examine the dynamic features of ionic interactions in SZIPs, emphasizing their structural advantages, controllable assembly, and ionic interaction characteristics. We further investigate the dynamic behavior driven by ionic interactions at the intramolecular, intermolecular, and supramolecular architecture levels. Finally, we discuss key challenges and future opportunities in this emerging field, aiming to advance the design and application of SZIPs.

## Graphical abstract

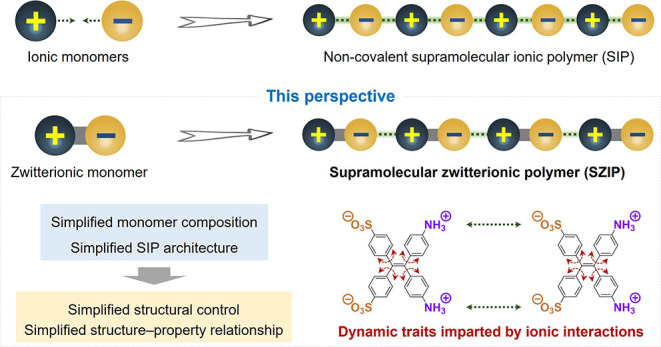

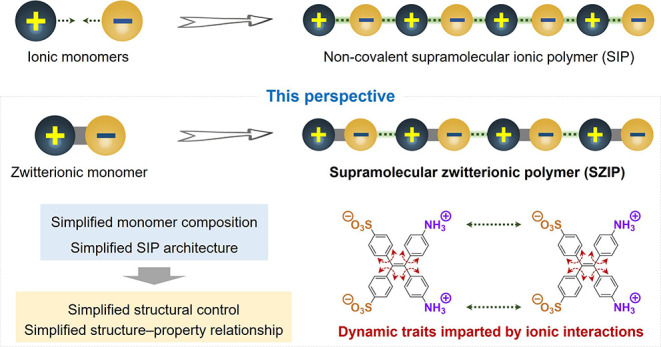

## 1 Introduction

Ionic aggregates represent one of the most ubiquitous forms of matter. Ionic interactions, characterized by substantial binding strength and notable dynamic properties, are distinct from both strong, irreversible covalent bonds and weak, transient supramolecular interactions.^1–3^ This unique combination renders ionic interactions highly significant in both the life sciences and materials science. In biological systems, ionic interactions are crucial for enzymatic catalysis, membrane stability, ion channel function, and antibody–antigen binding, serving as foundational elements for organization and regulation within living organisms.^4–8^ In materials science, the versatile properties of ionic interactions have enabled the extensive application of supramolecular ionic polymers (SIPs) across various fields, including energy storage, packaging, healthcare, and chemical engineering.^9–13^

Based on the bonding characteristics of polymer chains, SIPs can be broadly classified into two primary categories (Figure 1): covalent SIPs and noncovalent SIPs. Covalent SIPs are characterized by covalent bonds within their polymer chains, which confer exceptional stability and mechanical performance akin to traditional polymers.^14–17^ However, the irreversible nature of covalent cross-links presents significant challenges for recycling and reprocessing. Moreover, ionic interactions in covalent SIPs are generally confined to pendant groups or side chains, thereby limiting their reversible and dynamic functionalities. The amorphous structure of these polymers further hinders the establishment of precise structure–property relationships. In contrast, noncovalent SIPs are predominantly formed through ionic interactions within their polymer chains.^18–20^ These materials typically exhibit well-defined crystalline structures, striking a balance between stability and dynamic behavior. As a result, noncovalent SIPs have attracted increasing interest for applications in electrolytes, ionic liquids, gels, and porous materials.^21,22^

**Figure 1:**
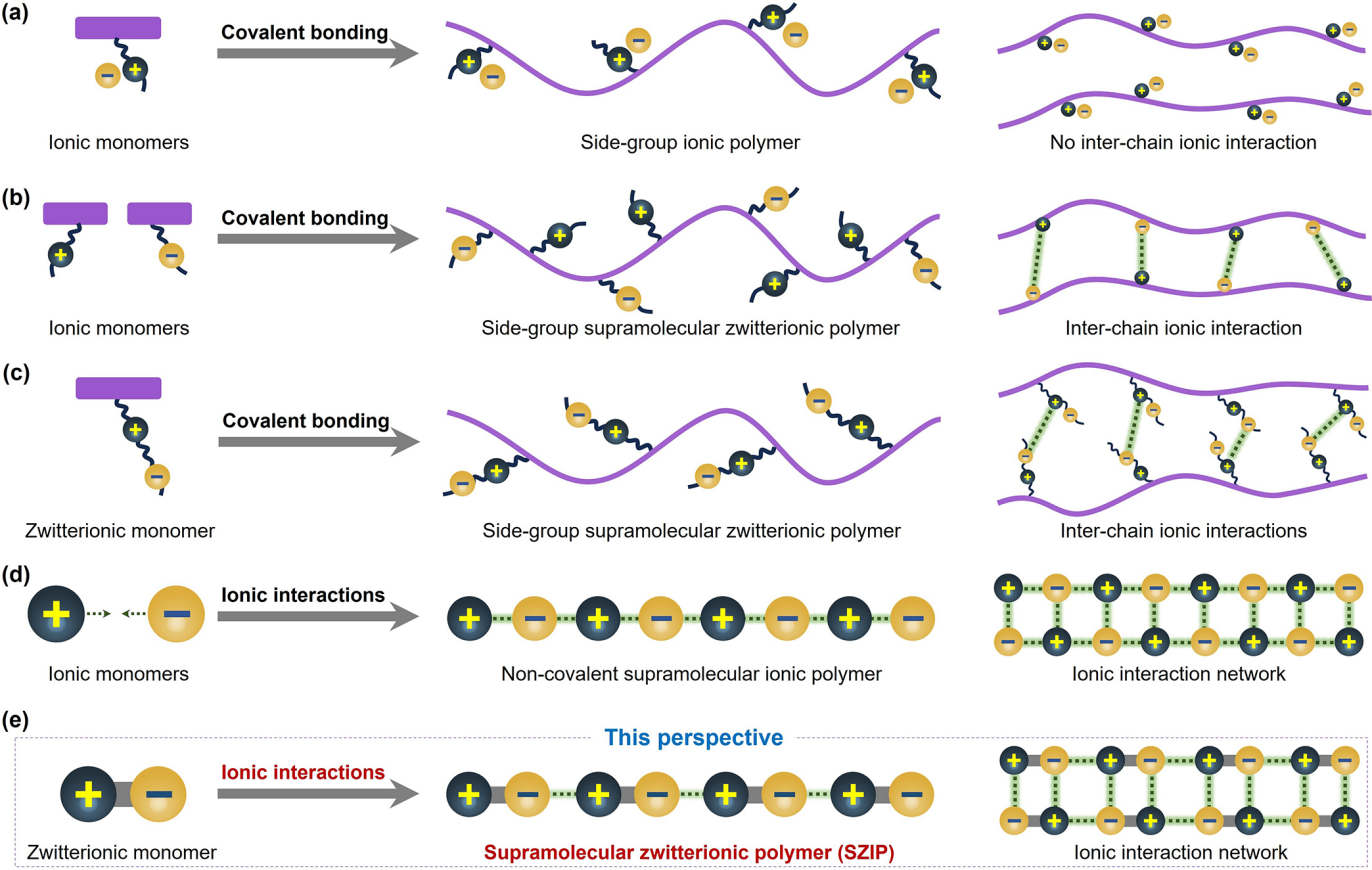
Schematic diagrams of different types of SIPs. (a) Schematic structure of an ionic polymer bearing only one type of ion. (b) Covalent SIPs obtained from the copolymerization of separated anionic and cationic monomers. (c) Covalent SIPs formed by a single-component zwitterionic monomer. (d) Noncovalent SIPs assembled from anionic and cationic double monomers. (e) SZIPs constructed from a single-component zwitterionic monomer.

The construction of SIPs is typically achieved through coassembly of binary monomers bearing complementary anionic and cationic moieties.^23–25^ This strategy has greatly broadened the scope of accessible monomers, thereby enriching both the structural diversity and functional complexity of SIP systems. Moreover, the relatively straightforward synthesis of monomers containing only anionic or cationic groups has substantially expanded the available monomer library. However, it also presents challenges. The inherently nondirectional and unsaturable nature of ionic interactions often leads to structural disorder, and the use of double monomers complicates both the synthesis and assembly process. A promising solution to these challenges is found in supramolecular zwitterionic polymers (SZIPs), which are constructed from single zwitterionic monomers.^26–29^ Zwitterionic monomers, such as molecules bearing both ammonium and carboxylate moieties, have long been recognized for their significance in both biological systems and materials science.^30–32^ Recently, our research group successfully synthesized a series of functional SZIPs based on zwitterionic monomers, showing dynamic traits imparted by ionic interactions.^33–35^ However, the systematic exploration of SZIPs as a distinct class has remained relatively underdeveloped. In SZIPs, as in all SIPs, ionic interactions are fundamental to assembly, structure, and function. These interactions are strong yet reversible, conferring the dynamic behavior typical of supramolecular polymers. Critically, the zwitterionic strategy simplifies both synthesis and structural modulation, thus creating an ideal model for probing dynamic ionic interactions. Research on dynamic materials, including molecular machines, switches, and rotors,^36–39^ is well-established; however, analyzing dynamics in conventional SIPs is notably complex. This complexity arises from the nondirectional and unsaturable nature of ionic interactions, which is exacerbated in binary monomer systems. Therefore, SZIPs offer a structurally simplified and highly valuable platform for in-depth studies of dynamics in SIPs.

Although zwitterionic species have been recognized for a long time, SZIPs have yet to be systematically defined in the literature, and there is currently no comprehensive review of these materials. Building upon our recent advancements in TPE-based SZIPs, this perspective seeks to establish SZIPs as a distinct class of supramolecular polymers. We begin by defining SZIPs in relation to covalent SIPs and conventional noncovalent SIPs, highlighting their unique assembly through robust ionic interactions between zwitterionic monomers. Subsequently, we analyze the characteristics of ionic interactions, with a focus on their combined strength and reversibility. Next, we explore how these ionic interactions confer dynamic properties to SZIPs at three distinct levels: intramolecular, intermolecular, and supramolecular architecture. Finally, we discuss future research opportunities for SZIPs, emphasizing the structural simplicity and dynamic behavior facilitated by the zwitterionic strategy. By elucidating the unique properties of SZIPs, this perspective aims to inspire further research and innovation across life sciences, materials science, and related fields.

## 2 Definition and Controlled Assembly of SZIPS

### 2.1 Definition and Structural Features

Ionic polymers are a class of polymers bearing ionic groups, which may be directly attached to the backbone or pendant from it (Figure 1a). Unlike conventional ionic polymers that carry only one type of charged species, zwitterionic polymers incorporate both cationic and anionic groups within their structure (Figure 1b,c).^40–43^ When ionic polymers engage in supramolecular interactions through ionic motifs, they can be classified as SIPs. Based on the bonding nature of the polymer chains, SIPs are broadly divided into two main categories: covalent SIPs and noncovalent SIPs. As previously indicated, covalent SIPs feature covalent bonding within the polymer chains. In contrast, noncovalent SIPs are formed primarily through ionic interactions along the polymer backbone (Figure 1d,e).^44–46^ These ionic interactions provide a combination of stability and dynamic reversibility. Moreover, the nondirectional and unsaturable character of ionic bonding promotes the formation of extensive interchain ionic networks.^47–49^ The term “supramolecular zwitterionic polymers” (SZIPs) captures the distinctive structural characteristics of these materials. Within this context, SZIPs are a specific subclass of noncovalent SIPs, defined as supramolecular polymer chains assembled from zwitterionic monomers via ionic interactions.^33–35^

The zwitterionic nature of SZIPs offers several distinct structural advantages. First, using single-component zwitterionic monomers to form SZIPs could eliminate various uncontrollable factors present in the formation of most noncovalent SIPs based on the double monomer method. These factors include the need for a strictly controlled binding ratio and phase separation due to differences in the solubility of double monomers.^50–52^ As a result, this strategy enables the construction of polymers with simpler and more well-defined architectures, which not only facilitates structure–property investigations but also allows for the precise modulation of material structures and properties. Second, SZIPs exhibit intrinsic flexibility and dynamic reversibility, which are critical advantages arising from the ionic interactions within the polymer chains. While ionic interactions in covalent SIPs can contribute some degree of dynamic behavior, the covalent backbone inherently imposes rigidity and irreversibility. In contrast, the polymer chains in SZIPs are entirely stabilized by noncovalent ionic interactions, forming a three-dimensional supramolecular network with dynamic properties. This unique network enables reversible transitions between polymer states and discrete nanoscale molecular assemblies, endowing SZIPs with unparalleled flexibility and adaptability. These distinctive features position SZIPs as a promising class of materials with unique structural and functional attributes, paving the way for advanced applications in diverse fields.

### 2.2 Controlled Assembly of SZIPs

The zwitterionic strategy also allows for better control over their assembly. Through modulating self-assembly parameters, such as the polarity of the solvent used and the *cis–trans* isomeric configuration of the monomer, a diverse range of SZIPs can be constructed. To demonstrate this, we synthesized a zwitterionic monomer, namely TPE-2N2S (Figure 2), based on a TPE core functionalized with ammonium cations and sulfonate anions at the *para* positions of the phenyl rings. TPE was chosen due to its fluorescence activity, structural symmetry, ease of chemical modification, and the rotational freedom of its phenyl rings, which provide diverse molecular conformations and versatile optical properties. Through the self-assembly of TPE-2N2S in different solvent conditions, we successfully constructed two SZIPs—SZIP-1 and SZIP-2 (Figure 2a). Specifically, SZIP-1 adopts a side-by-side chain structure with dense interchain ionic interactions, whereas SZIP-2 forms a head-to-tail chain structure with fewer interchain ionic contacts. This demonstrates that even with a single zwitterionic monomer, structural diversity can be achieved through controlled assembly.

**Figure 2:**
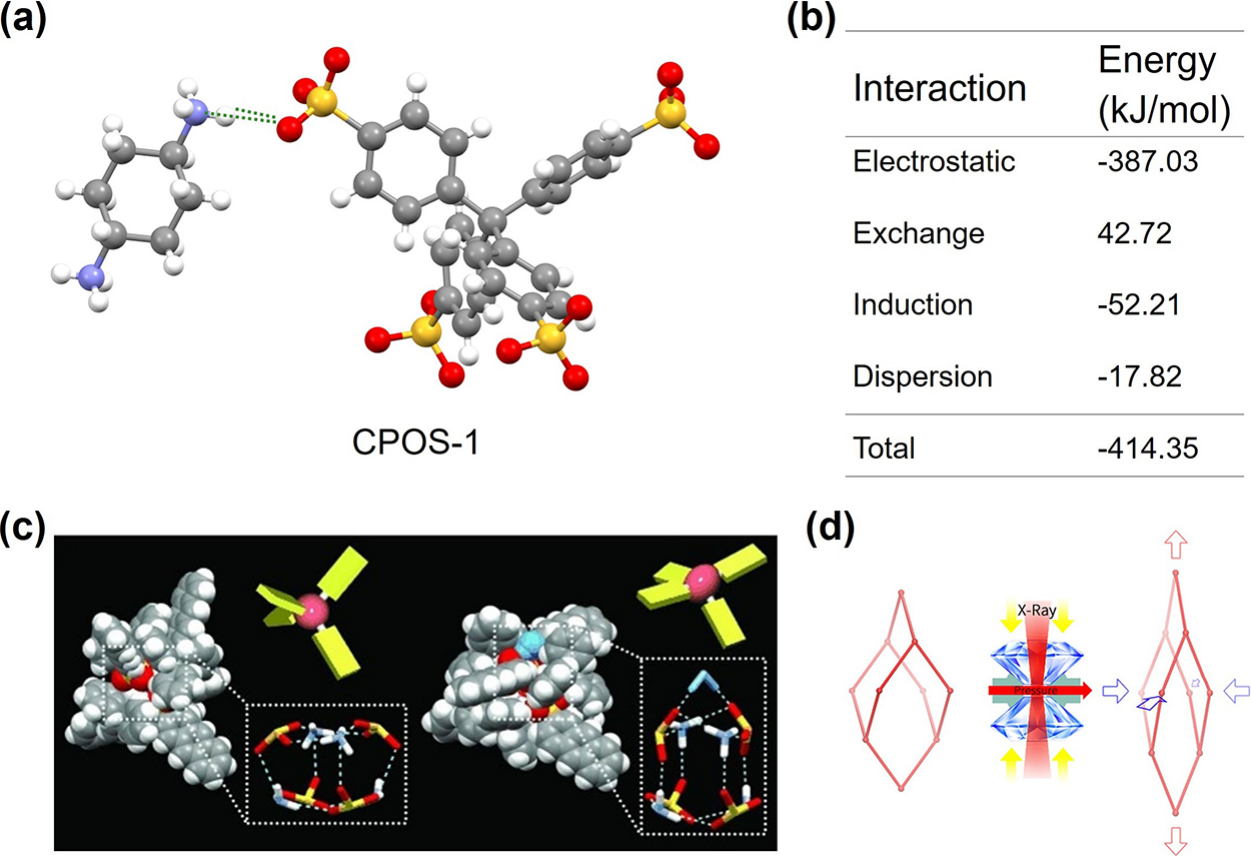
Controlled assembly of SZIPs. (a) Diverse SZIPs obtained from the same monomer by modulating the assembly conditions. (b) Distinct SZIPs achieved by controlling the *cis–trans* isomeric configuration of the monomer.

Building on this strategy, two isomeric monomers, namely TPE-2N2S-Z and TPE-2N2S-E, were synthesized to explore the influence of *cis–trans* isomerism. Under the same assembly conditions, they formed different SZIPs (namely SZIP-3 and SZIP-4, Figure 2b). The polymer structure differences between SZIP-1 and SZIP-2, as well as between the isomer-derived SZIP-3 and SZIP-4, revealed significant variations in chain packing architectures, porous structures, and dynamic properties. Notably, the distinct chain architectures of SZIP-1 and SZIP-2 led to pronounced differences in their photoresponsive behaviors, while the *cis–trans* isomerism in SZIP-3 and SZIP-4 further modulated their structural and functional attributes. These findings highlight the versatility and tunability of SZIPs, which will be explored in detail in subsequent sections.

## 3 Substantial Strength and Dynamic Character of Ionic Interactions

The nature of chemical bonding fundamentally determines the structural formation and material properties of polymers. Covalent bonds, characterized by high bond energies, require significant energy input to break, which is the fundamental reason for the high stability they impart to molecular structures.^53–55^ In contrast, supramolecular interactions, characterized by their lower bond energies and reversible nature, allow for monomers to form complex and ordered supramolecular architectures through a spontaneous assembly process.^56–63^ These interactions, including hydrogen bonding, van der Waals forces, π–π stacking, and hydrophobic effects, et al, exhibit relatively low strength, leading to reduced structural stability but enhanced reversibility.^64–68^ Among supramolecular forces, ionic interactions combine substantial strength with a dynamic nature, thereby distinguishing SIPs from both covalent polymers and other supramolecular assemblies.^69–72^

### 3.1 Substantial Strength of Ionic Interactions

The exceptional strength of ionic interactions can be quantified through computational studies. For example, Professor Ben’s group investigated the interaction energies of their organic ionic system, CPOS-1 (Figure 3a,b), using computational simulations.^72^ Interactions between organic acids and bases encompass electrostatic, exchange, induction, and dispersion forces. The total interaction energy of CPOS-1 was calculated to be 414.35 kJ/mol, a value reflecting the combined contributions of hydrogen bonding and ionic interactions. To isolate the ionic contribution, the researchers developed a neutralized model system where charged groups were replaced with neutral analogs. In this modified system, the electrostatic interaction energy decreased dramatically to 23.63 kJ/mol, while other interaction terms remained largely unchanged. The total interaction energy in the neutral system was approximately 50.94 kJ/mol, primarily attributed to hydrogen bonding. By comparing the charged and neutral systems, the ionic interaction energy was estimated to be 363.40 kJ/mol, substantially exceeding the strength of typical supramolecular interactions, such as hydrogen bonding, dipole–dipole interactions, and π–π stacking. This high interaction strength underpins the stability of ionic assemblies while allowing for dynamic reorganization under external stimuli.

**Figure 3:**
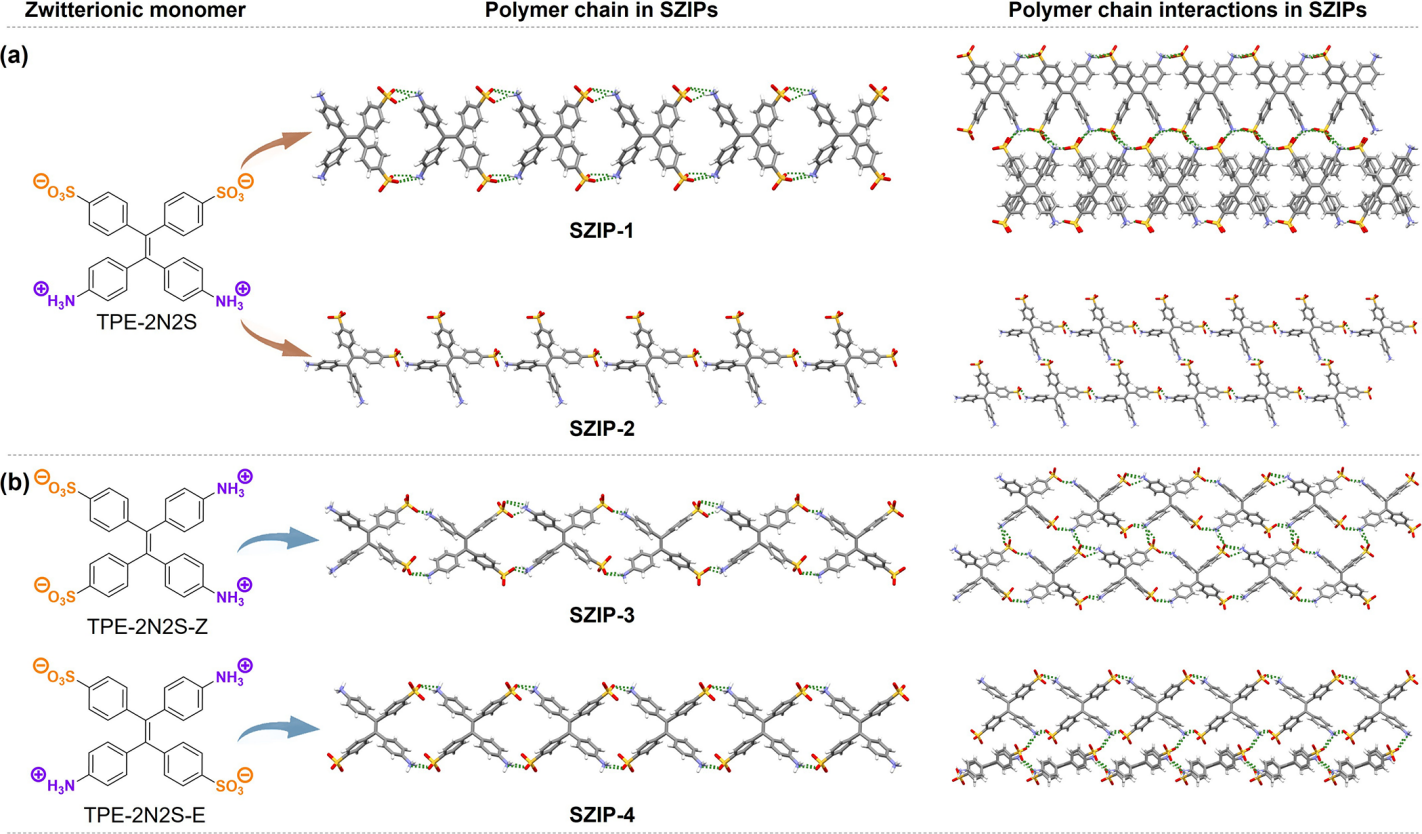
Substantial strength and dynamic character of ionic interactions. (a, b) Molecular fragments of CPOS-1 and the corresponding interaction energy between the organic acid and base. Reproduced with permissions from ref 72. Copyright 2024 Royal Society of Chemistry. (c) Water-induced crystal distortion: a highly symmetrical, cube-like hydrogen-bonded tetrahedral cluster composed of 2-AS and TPMA (left), and the resulting distorted cluster incorporating a water molecule (right). Reproduced with permissions from ref 73. Copyright 2013 Wiley-VCH. (d) Colossal negative linear compressibility behavior observed in CPOS-1. Reproduced with permissions from ref 74. Copyright 2020 American Chemical Society.

### 3.2 Dynamic Character of Ionic Interactions

The dynamic nature of ionic interactions is exemplified in several systems. For instance, Tohnai and colleagues developed diamond-type porous organic salts (d-POSs) based on ionic interactions between 2-anthracenesulfonic acid (2-AS) and *trans*-1,2-bis­(4-pyridyl)­ethylene (TPMA).^73^ These materials exhibit reversible guest molecule encapsulation and release under varying environmental conditions. At room temperature, d-POSs can encapsulate guest molecules, whereas under high humidity, they transition to a nonporous structure (NP-1, Figure 3c). Structural analysis revealed that water molecules penetrate the crystalline core, inducing the rotation of sulfonate groups. This rotation facilitates the formation of hydrogen bonds with water, resulting in core distortion and linear alignment of two anthracene units. The aromatic shielding effect subsequently prevents further water ingress, stabilizing the distorted configuration and inducing fluorescence changes that are detectable under optical analysis.

Another striking example of dynamic ionic behavior is the rare phenomenon of negative linear compressibility (NLC), in which a material expands along one axis under hydrostatic pressure. Professor Ben’s group reported that CPOS-1 exhibits significant NLC behavior (*K*_c_ = −90.7 T/Pa along the *c*-axis) during high-pressure X-ray diffraction experiments (Figure 3d).^74^ This unusual property stems from a flexible “supramolecular spring” structure formed through charge-enhanced N–H^+^···^–^O–S hydrogen bonds between anionic sulfonate and cationic ammonium groups. Under pressure, the ionic interactions and hydrogen bonds work cooperatively to allow structural reorganization, resulting in anisotropic expansion. This behavior highlights the inherent flexibility and adaptability of ionic networks in response to external mechanical stimuli.

The combination of substantial strength and dynamic reversibility makes ionic interactions a cornerstone of SZIPs design. Their strong yet reversible nature enables the formation of stable supramolecular networks that can undergo dynamic structural transitions under external stimuli, such as solvent changes, humidity, or pressure. These properties not only enhance the functional versatility of SZIPs but also differentiate them from other supramolecular systems, establishing them as a unique platform for advanced material applications.

## 4 Dynamic Nature of SZIPS

Building on the established structural features of SZIPs and the unique synergy of strength and dynamic behavior enabled by ionic interactions, we now turn our attention to the central theme of this perspective: the dynamic properties conferred to SZIPs through ionic interactions. These dynamics manifest across three hierarchical levels—intramolecular, intermolecular, and supramolecular architectural—each unveiling distinct functional characteristics and contributing to the versatile behavior of SZIPs.

### 4.1 Intramolecular Dynamics Activated by Strong Ionic Interactions

In covalent polymers, intramolecular dynamics typically involve the breaking and formation of covalent bonds, often accompanied by conformational changes that enable the system to reach a new thermodynamic equilibrium.^75,76^ In conventional supramolecular polymers, which rely on weak noncovalent interactions, monomer assembly generally occurs without significant changes to the intrinsic molecular conformation, as monomers retain their thermodynamically stable structure from the single-molecule state.^77,78^ This raises a key question: can the strong ionic interactions in SZIPs induce conformational changes in monomers during the assembly process? The zwitterionic strategy and the photofunctional TPE-2N2S monomer provide an ideal mode to explore this question. First, the conformation of TPE-2N2S was analyzed in the single-molecule state.^33^ Figure 4a,b shows the energy profile (*E*_R_) associated with the dihedral angle (θ_Ph2–3_) rotation between the phenyl rings P2 and P3. A smaller θ_Ph2–3_ corresponds to higher energy due to steric repulsion, while larger θ_Ph2–3_ values eventually destabilize the conjugated TPE framework. The thermodynamically stable conformation occurs at θ_Ph2–3_ = 55°, corresponding to the lowest energy for the isolated monomer.

**Figure 4:**
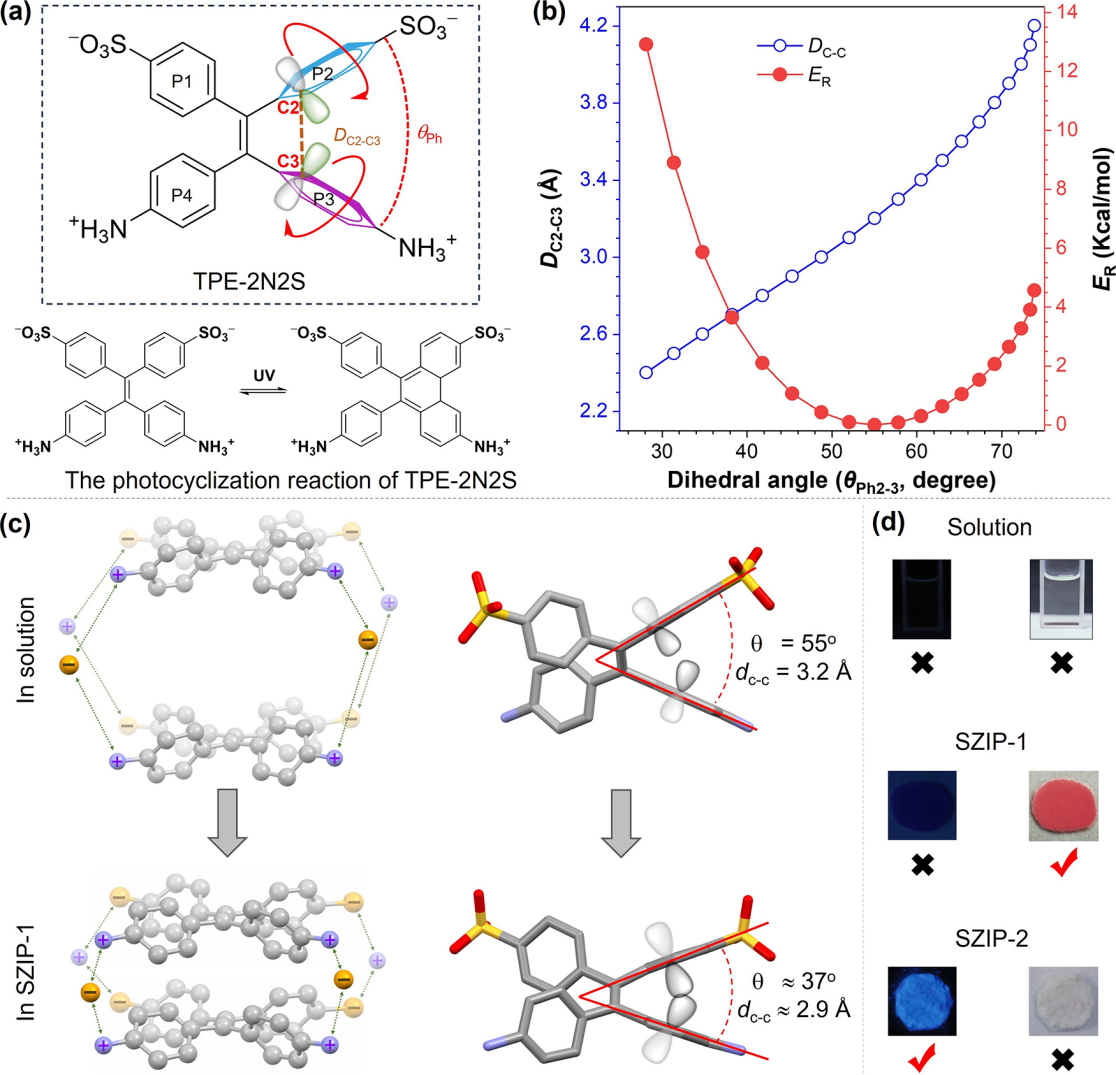
Intramolecular dynamics activated by strong ionic interactions. (a) Molecular structure of monomer TPE-2N2S and schematic of the photocyclization reaction. (b) Plot of *D*_C2–C3_ and the relative energy (*E*_R_) versus θ_Ph2–3_. (c) Schematic of the assembly process from TPE-2N2S to SZIP-1. (d) Photochromic and fluorescence activities under different states. Reproduced with permissions from ref 33. Copyright 2024 ELSEVIER.

In contrast, analysis of the crystal structures of SZIP-1 and SZIP-2 revealed significant differences in θ_Ph2–3_ (Figure 4d). In SZIP-1, θ_Ph2–3_ is compressed to 37°, representing a high-energy state for the monomer, while in SZIP-2, θ_Ph2–3_ is 58°, close to the thermodynamically stable conformation of the molecule at the single-molecular state. These differences highlight two key aspects of intramolecular dynamics in SZIPs. The first is the compression of monomer conformation. During SZIP-1 assembly, strong intermolecular ionic interactions compress the TPE-2N2S monomer, forcing it into a high-energy conformation. This compression sacrifices the monomer’s individual thermodynamic stability to achieve the overall thermodynamic stability of the supramolecular polymer architecture (Figure 4c). The second is the activation of photocyclization reactivity. The conformational dynamics in SZIP-1 also influence photoresponsive behavior. In diarylethenes, the distance between reactive carbon atoms (*D*_C–C_) critically affects photocyclization.^79–81^ For TPE-2N2S, the *D*_C–C_ decreases from 3.2 Å (single-molecule state) to 2.9 Å in SZIP-1, activating photocyclization. It has been experimentally confirmed that SZIP-1 exhibits photochromic activity, while SZIP-2, with a relaxed conformation and larger *D*_C–C_, does not exhibit such activity. The compressed conformation in SZIP-1 favors photocyclization but suppresses fluorescence, while the relaxed conformation in SZIP-2 inhibits photocyclization, favoring fluorescence emission. These findings demonstrate that ionic interactions not only regulate monomer conformation during assembly but also activate emergent dynamic properties, such as photocyclization-induced photochromism in the aggregate state.

### 4.2 Intermolecular Dynamics Driven by Strong Ionic Interactions

Dynamic intermolecular motion is another hallmark of SZIP behavior. As shown in Figure 5a,b, the TPE-2N2S monomer exhibits distinct fluorescence activity under UV light depending on its assembly state.^34^ The pristine crystalline powder emits deep-blue fluorescence (PLQY = 14.2%), while grinding transforms it into an amorphous state with red-shifted light-blue fluorescence (PLQY = 5.1%). Over time, the amorphous state spontaneously evolves into a recovered aggregate state with dim deep-blue fluorescence (PLQY = 0.5%).

**Figure 5:**
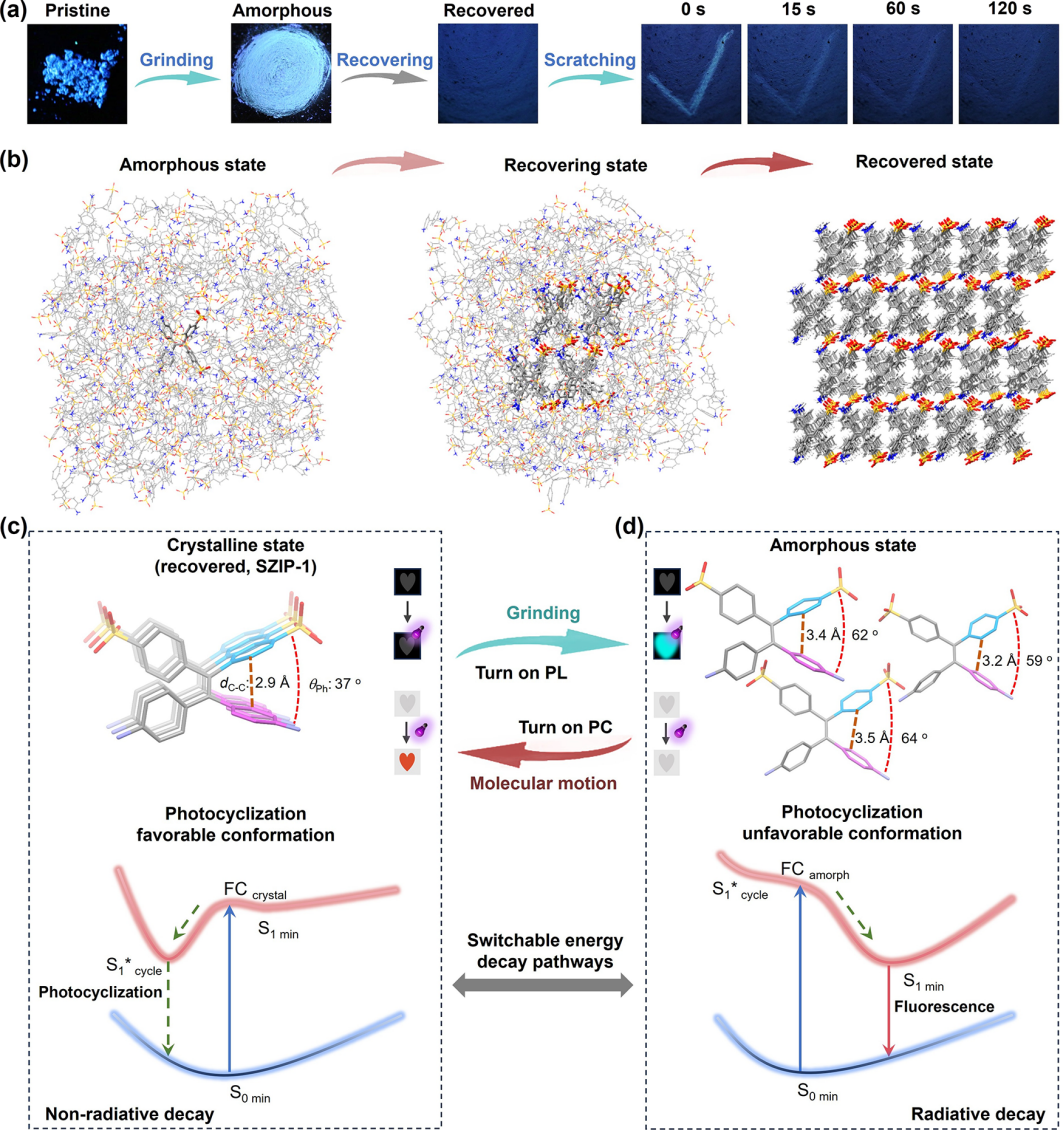
Intermolecular dynamics driven by strong ionic interactions. (a) Transition in PL signal of zwitterionic aggregate after grinding and self-recovery. (b) MD simulation models representing the amorphous state, recovering state, and recovered state. (c) Depiction of the uniform molecular packing of TPE-2N2S in the crystalline γ phase, highlighting a conformation conducive to photocyclization, and the schematic representation of the nonradiative decay pathway resulting from the photocyclization reaction upon UV irradiation. (d) Illustration of disordered packing of TPE-2N2S exhibiting conformations that are unfavorable for photocyclization in the amorphous state, and the schematic representation of the radiative decay pathway resulting from suppressed photocyclization reactivity upon UV irradiation. Reproduced with permissions from ref 34. Copyright 2025 Oxford University Press.

This dynamic transition between different states is reversible. In detail, scratching the recovered sample creates a transient light-blue emission pattern that fades within 120 s, indicating molecular reorganization within the aggregate. Powder X-ray diffraction (PXRD) patterns confirm that the recovered state differs structurally from the pristine crystalline state but matches the ordered structure of SZIP-1. In contrast, nonzwitterionic analogs (e.g., TPE-0N4S and TPE-4N0S) lack this recovery behavior, transitioning irreversibly to an amorphous state upon grinding. These results suggest that ionic interactions are the driving force behind the reversible recovery process.

This behavior is explained by the interplay between intermolecular ionic interactions and conformational dynamics (Figure 5c,d). In the crystalline SZIP-1 structure, ionic interactions stabilize the compressed monomer conformation (θ_Ph2–3_ = 37°), favoring photocyclization-induced photochromism and suppressing fluorescence. Upon grinding, the crystalline structure breaks down, weakening intermolecular ionic interactions and allowing monomers to adopt relaxed conformations with larger θ_Ph2–3_ and *D*_C–C_ values, favoring fluorescence emission. Over time, strong ionic interactions drive the reorganization of the amorphous state back into the ordered crystalline structure of SZIP-1, reactivating photochromism and suppressing fluorescence. These reversible intermolecular dynamics demonstrate the robustness and adaptability of ionic interactions in SZIP aggregates.

### 4.3 Supramolecular Architecture Dynamics Enabled by Strong Ionic Interactions

At the supramolecular architecture level, ionic interactions also enable dynamic behavior, such as flexible porosity. This is exemplified by the crystal structure of SZIP-4, which contains a porous framework stabilized by dimethyl sulfoxide (DMSO) molecules (Figure 6a).^35^ Immersion in methanol induces structural transformation, as confirmed by thermogravimetric (TGA) and PXRD analysis, which shows the removal of DMSO, activating the porous structure (Figure 6b,c). Gas adsorption measurements reveal a micropore volume of 0.2281 cm^3^/g and an average pore diameter of 0.6143 nm. Remarkably, reimmersion in DMSO restores the original crystal structure, demonstrating reversible porosity. Solvent-induced transformations were systematically studied (Figure 6d). Solvents such as methanol and ethanol induce reversible changes, while water causes irreversible pore collapse, and others (e.g., acetone, THF) induce no change.

**Figure 6:**
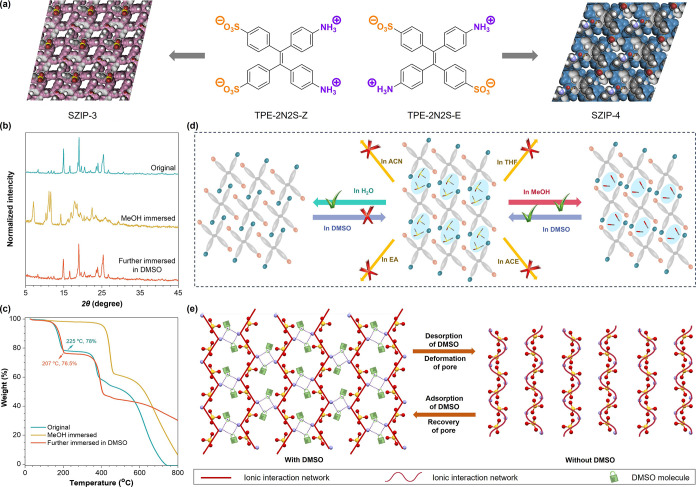
Supramolecular architecture dynamics enabled by strong ionic interactions. (a) Distinct SZIPs and their porous structures achieved by controlling the *cis–trans* isomeric configuration of the monomer. (b) The PXRD patterns of the original sample of SZIP-4, after immersion in methanol, and after reimmersion in DMSO of the immersed sample in methanol. (c) The TGA curves of SZIP-4, after immersion in methanol, and after reimmersion in DMSO of the immersed sample in methanol. (d) The proposed schematic diagram of flexible pore characteristics in SZIP-4 and the selective release of DMSO molecules upon immersion in different solvents. (e) Ionic interaction network and ionic-DMSO interactions in SZIP-4, and the schematic diagram of simplified ionic interactions and ionic-DMSO interaction in SZIP-4. Reproduced with permissions from ref 35. Copyright 2024 American Chemical Society.

Structural analysis (Figure 6e) reveals that DMSO molecules act as cross-linkers, stabilizing the SZIP-4 framework. Upon removal of the DMSO, ionic interactions between TPE-2N2S-E monomers allow the framework to collapse into a denser configuration, which can be reversed upon DMSO reintroduction. This flexibility arises from the polymer-like nature of the ionic network, where DMSO molecules function as dynamic stabilizers. This reversible structural deformation highlights the dual role of ionic interactions: providing stability under mechanical or chemical stress while enabling dynamic flexibility for repeated transformations.

## 5 Conclusion and Perspectives

This perspective has established SZIPs as a distinct and promising class of materials, characterized by self-assembly from single-component zwitterionic monomers via strong yet reversible ionic interactions. The zwitterionic strategy effectively overcomes key limitations of conventional double monomer SIPs, notably the synthetic complexity and structural disorder, by offering a streamlined pathway to well-defined and tunable supramolecular polymers. Through the exemplar system of TPE-2N2S, we have demonstrated that ionic interactions in SZIPs confer remarkable dynamic properties across multiple hierarchical levels: intramolecularly, by manipulating monomer conformation to activate emergent functions like photocyclization-induced photochromism; intermolecularly, by enabling reversible structural recovery and fluorescence switching; and supramolecular architecture, by facilitating stimuli-responsive porosity and framework transformations. This multilevel dynamics, underpinned by the unique combination of strength and reversibility inherent to ionic interactions, positions SZIPs as a versatile platform for constructing intelligent, adaptive, and functional materials.

Looking forward, the field of SZIPs, though nascent, presents several compelling avenues for future research. First, more efforts should be made to expand the monomer library and functionality. The current exploration of SZIPs relies on a limited set of zwitterionic monomers. A paramount challenge and opportunity lies in the de novo design and synthesis of a diverse library of zwitterionic building blocks. Compared to molecules bearing only anionic or cationic groups, the structure of zwitterionic monomers is more complex, which may pose synthetic challenges. Future efforts should focus on incorporating various functional cores and tuning the nature and spatial arrangement of the ionic groups. This will significantly broaden the structural diversity and functional scope of SZIPs, enabling applications in catalysis, sensing, biomedicine, and beyond. Second, the dynamic mechanisms should be further deciphered and harnessed. While we have begun to unravel the dynamic behavior of SZIPs, a deeper, quantitative understanding of the kinetics and thermodynamics of their ionic interactions is crucial. Advanced in situ and operando characterization techniques, coupled with sophisticated theoretical modeling, are needed to probe the real-time breakage and reformation of ionic linkages under external stimuli. Establishing precise structure–dynamics–property relationships will be key to rationally designing SZIPs with predictive and programmable responses for applications such as self-healing materials, adaptive membranes, and recyclable plastics. And third, the gap to macroscopic applications should be bridged. The exceptional dynamics of SZIPs have been predominantly demonstrated at the molecular and microscopic levels. The next critical step is to translate these properties into macroscopic materials and devices. This entails processing SZIPs into robust bulk materials, thin films, fibers, and coatings without compromising their dynamic character. Exploring their performance in practical settings—such as solid-state electrolytes with self-healing interfaces, smart separation membranes with tunable porosity, or recyclable adhesives—will be essential to validate their technological potential and drive the field from fundamental concept to tangible application.

In conclusion, SZIPs represent a paradigm shift in supramolecular polymer science, leveraging the power of zwitterionic motifs to create structurally simplified yet functionally complex systems. By offering unparalleled control over dynamic behavior across multiple scales, they open a new frontier for designing responsive and sustainable materials. We anticipate that this perspective will inspire concerted efforts to explore the vast chemical space and application potential of this emerging and dynamic family of supramolecular polymers.
